# Vitamin D and Omega-3 Supplementation for Emotional and Behavioral Dysregulation in Autism Spectrum Disorders: A Systematic Review

**DOI:** 10.3390/jcm15020745

**Published:** 2026-01-16

**Authors:** Marta Berni, Giulia Mutti, Raffaella Tancredi, Filippo Muratori, Sara Calderoni

**Affiliations:** 1 University of Florence, 50121 Florence, Italy; marta.berni1@unifi.it; 2 IRCCS Stella Maris Foundation, 56128 Pisa, Italy; giulia.mutti@fsm.unipi.it (G.M.); raffaella.tancredi@fsm.unipi.it (R.T.); 3 Stella Maris Mediterraneo Foundation, 85032 Chiaromonte, Italy; filippo.muratori@fsm.unipi.it; 4 Department of Clinical and Experimental Medicine, University of Pisa, 56126 Pisa, Italy

**Keywords:** autism spectrum disorder, vitamin D, omega-3, supplementation, behavioral dysregulation, emotional dysregulation

## Abstract

**Background/Objectives:** Emotional dysregulation (ED) is emerging as a major contributor to functional impairment in Autism Spectrum Disorder (ASD). Although effective behavioral interventions exist, pharmacological treatments remain constrained by side effects and variable tolerability. Given their neurobiological roles that include neurotransmission, inflammation, and neuroplasticity, vitamin D and omega-3 polyunsaturated fatty acids (PUFAs) have been identified as promising candidates for modulating emotional and behavioral dysregulation. This systematic review aimed to evaluate the efficacy of combined vitamin D and omega-3 supplementation in improving emotional and behavioral regulation in individuals with ASD. **Methods:** This review was conducted in accordance with PRISMA guidelines. Included studies were English peer-reviewed studies involving participants with ASD that assessed combined vitamin D and omega-3 suppleupplementation with outcomes related to emotional or behavioral dysregulation. The search was restricted to 2015–2025 to ensure inclusion of recent, methodologically consistent studies and to minimize heterogeneity in diagnostic criteria and supplementation protocols. **Results:** Of 649 records initially screened, 3 studies met inclusion criteria: one randomized controlled trial, one observational study, and one case report, involving participants ranging from early childhood to young adulthood. Across studies, combined supplementation was associated with improvements in irritability, hyperactivity, agitation, and self-injurious behaviors. These clinical effects were accompanied by specific biochemical changes, including reductions in the AA/EPA ratio, increases in serum 25(OH)D and omega-3 indices, and decreased urinary levels of HVA and VMA. **Conclusions:** This review indicates that co-supplementation with vitamin D and omega-3 fatty acids may exert preliminary beneficial effects on emotional and behavioral dysregulation in individuals with ASD, potentially through anti-inflammatory and neuroregulatory mechanisms. However, the available evidence remains limited due to a small number of studies, their modest sample size, and methodological heterogeneity. Further, biomarker-driven randomized studies are needed to confirm efficacy and delineate optimal dosing strategies for application in clinics.

## 1. Introduction

### 1.1. Autism Spectrum Disorder and Emotional Dysregulation

Autism Spectrum Disorder (ASD) is a heterogeneous neurodevelopmental condition that emerges at an early developmental age, characterized by social communication and interaction impairments, as well as repetitive behaviors and restricted interests [1]. Autistic people frequently experience additional behavioral challenges and comorbidities, such as difficulties in emotional regulation (for two recent meta-analyses, see [2,3]). Although Emotional Dysregulation (ED) is not a core clinical criterion for ASD diagnosis, it is a significant contributor to functional impairment and clinical referral and seems closely linked to core symptoms of the disorder [4,5]: autistic people often have difficulty in identifying and managing emotions, suppressing emotional responses, delaying gratification, and tolerating transitions [6]. Emotion dysregulation and/or irritability have been conceptualized as the failure to regulate emotions appropriately and effectively [7] and a state of reduced control over temper or an excessive response to stimuli, respectively [8]. The importance of investigating ED in ASD is further underscored by the strong association between ED and the high prevalence of co-occurring psychiatric conditions, including anxiety disorders, attention-deficit/hyperactivity disorder (ADHD), mood disorders, and disruptive behavior disorders [9,10,11]. These comorbidities significantly increase the probability and the severity of irritability, affective instability, and behavioral dysregulation, exacerbating functional impairment and raising rates of clinical referral [5]. There is still no widely accepted definition of ED, despite growing interest in it as a transdiagnostic risk factor for mental health, highlighting its potential as a target for customized therapeutic interventions [12]. Bunford, Evans & Wymbs [13] offer the most prevalent definition of emotion dysregulation, which describes it as “an individual’s inability to exercise any or all aspects of the modulatory processes involved in emotion regulation, to such a degree that this inability results in functioning meaningfully below baseline”. As commonly conceptualized, ED is typically characterized by three main features: exaggerated emotional responses according to social norms, rapid and uncontrollable emotional fluctuations, and maladaptive attention to emotional stimuli. Furthermore, ED includes not only difficulties in recognizing and accepting emotions but also impairments in selecting adaptive coping strategies [14,15]. Despite the increasing awareness of ED impact in ASD [2,16,17], there are few validated interventions to enhance emotional regulation in individuals with ASD. Specifically, non-pharmacological and pharmacological approaches were proposed to manage ED. Non-pharmacologic strategies typically involve the identification of potential triggers for behavioral and environmental interventions to reduce the frequency and intensity of ED episodes [18]. Moreover, cognitive-behavioral interventions yielded promising results for strengthening emotional regulation in children with ASD and adolescents [19]. If ED remains a significant challenge to the ASD individual’s functioning despite the implementation of non-pharmacological approaches, then pharmacological intervention could be considered as part of a multimodal treatment plan. Although there is no pharmacological treatment directed toward ED among individuals with ASD, several medications have been investigated for psychiatric comorbidities associated with ED, although evidence is limited or inconsistent. These include atypical antipsychotics [20]; ADHD medications, especially if ED co-occurs with significant ADHD symptoms [21]; mood stabilizers such as valproate or lamotrigine [22]; glutamatergic modulators [23]; and neuropeptides such as oxytocin or secretin [24]. Risperidone and aripiprazole are antipsychotic drugs FDA (Food and Drug Administration) approved as antipsychotic medications specifically for managing irritability, which frequently is manifested through uncontrolled anger, frustration, and distress with or without aggression [25]. Despite several studies, including clinical trials, providing evidence for the beneficial effects of risperidone and/or aripiprazole treatment in the management of ED in individuals with ASD, the possible onset of acute and chronic side effects should also be carefully considered [26].

### 1.2. Nutritional Supplementation in ASD

Concerns regarding the tolerability of pharmacological treatments, together with the need for long-term management of emotional dysregulation, have contributed to growing interest among families and clinicians in the use of Complementary and Alternative Medicine (CAM) as an intervention strategy for autism. According to epidemiological studies, CAM utilization rates can reach up to 74% in families with an autistic child [27,28], indicating that caregiver interest is widespread and its general perceived acceptability is high. Among the various biological modalities within CAM, the most commonly adopted are nutritional interventions, such as specialized diets and vitamin supplementation [29]. Their widespread use has been driven by the perception of safety and naturalness, with particular emphasis on supplementation with polyunsaturated omega-3 fatty acids and vitamin D, which are essential for brain function and neurobehavioral regulation [30,31,32]. Of specific interest, omega-3 PUFAs, such as α-linolenic acid (ALA), eicosapentaenoic acid (EPA), and docosahexaenoic acid (DHA), are critical components of neuronal membranes that modify membrane fluidity, regulating neurotransmission, neurogenesis, synaptic plasticity, and neuronal survival. These essential fatty acids are found mainly in natural foods and are predominantly available through diet, from green leafy vegetables, chia seeds, and plant oils or deep-sea fish oil [33]. Vitamin D is a neuroactive steroid, which exerts broad regulatory effects beyond calcium-phosphate homeostasis. It is primarily derived from ultraviolet B (UVB) exposure and is also supplemented with diet. Cholecalciferol (vitamin D3) and ergocalciferol (vitamin D2), the main precursors of vitamin D, undergo sequential hepatic and renal hydroxylation to produce calcitriol (1,25-dihydroxy vitamin D, 1,25(OH)2D), its active form. Vitamin D regulates neuronal differentiation, axonal connectivity, neurotransmitter synthesis, and neuroimmune modulation, among numerous effects on brain development and function [34]. Vitamin D is measured by serum concentration of 25(OH)D, but optimal levels have not been defined, and consensus has not been reached: the Institute of Medicine [35] defines sufficiency as levels ≥ 20 ng/mL, whereas the Endocrine Society [36] recommends a higher threshold, with sufficiency defined as ≥30 ng/mL. Omega-3 and omega-6 PUFA status is measured by the reduction in plasma fatty acid concentration (recent intake) and in erythrocytes that shows long-term status. The omega-3 Index, defined as the combined percentage of eicosapentaenoic acid (EPA) and docosahexaenoic acid (DHA) in erythrocyte membranes, serves as a biomarker of cardiovascular health, with values ≥ 8% considered optimal [37]. Erythrocyte omega-3 levels typically range from 4% to 12%, while omega-6 fatty acids, such as linoleic (LA) and arachidonic acid (AA), account for 20–30% of total erythrocyte fatty acids. The omega-6 to omega-3 ratio, (preferably less than 4:1) serves as a determinant of the ratio of these polyunsaturated fatty acid classes [38]. Recent evidence indicates a synergistic effect of omega-3 PUFAs and vitamin D in modulating systemic inflammation and immune function. Co-supplementation with vitamin D and omega-3 PUFAs, often known as VIDOM therapy, has demonstrated beneficial effects in conditions linked to chronic inflammation, including ASD [39]. Experimental studies have shown that vitamin D deficiency can alter PUFA metabolism, increasing AA levels and Δ5-desaturase activity, an important enzyme in the biosynthesis of omega-3 and omega-6 [40]. Conversely, omega-3 supplementation may enhance vitamin D activation by inhibiting catabolic enzymes (24-hydroxylase) and upregulating activating enzymes (1α-hydroxylase), increasing circulating 1,25(OH)2D levels [41]. These findings provide a mechanistic explanation for the synergistic anti-inflammatory and neuroprotective effects of combined supplementation. Vitamin D may be administered orally or intramuscularly, with recommended supplementation varying between 400 and 2000 IU/day according to age, body weight, disease status, and ethnicity [42]. Toxicity is unusual and is mostly associated with hypercalcemia, but dose adjustments need to be made cautiously. Importantly, individuals with ASD may present with a decreased serum response due to supplementation, necessitating routine monitoring of serum levels of 25(OH)D to ensure that therapy is efficacious [43]. According to the National Institutes of Health’s (NIH) recommendations based on adult cohorts, weight-adjusted omega-3 fatty acid (EPA, DHA) supplementation typically ranges from approximately 30 mg/kg/day for cardiovascular maintenance to 50 mg/kg/day in order to optimize the Omega-3 Index [44]. Higher doses, up to 100 mg/kg per day, may be prescribed under medical supervision for the treatment of hypertriglyceridemia [45]. A growing body of clinical research has investigated the effects of combined omega-3 and vitamin D supplementation using heterogeneous dosing strategies, which complicates cross-study comparisons. Nevertheless, recent systematic reviews have highlighted potential beneficial effects in individuals with ASD, particularly with respect to social interaction and behavioral outcomes (e.g., [46]). Regarding emotional and behavioral dysregulation, this includes emotional difficulties (e.g., [47,48]), irritability (e.g., [49]), and broader behavioral problems (e.g., [50]), but remains heterogeneous. Despite these encouraging findings, it is important to consider the safety and tolerability of these supplements. Although vitamin D and omega-3 fatty acids are generally considered safe, high-dose vitamin D supplementation may lead to gastrointestinal symptoms, hypercalcemia, or, in rare cases, nephrolithiasis, particularly in individuals with impaired vitamin D metabolism [51]. Omega-3 fatty acids are generally well tolerated but can lead to gastrointestinal symptoms or mild effects on platelet aggregation in some individuals [52]. These safety considerations highlight the need for individualized dosing strategies and regular biochemical monitoring, especially when these nutrients are employed as long-term adjunctive intervention in ASD.

Previous systematic reviews have investigated vitamin D and omega-3 supplementation in ASD [39,46]; however, emotional and behavioral dysregulation has often been addressed as a secondary or heterogeneous outcome rather than as a primary focus.

## 2. Materials and Methods

This study was performed according to PRISMA guidelines (Preferred Reporting Items for Systematic Reviews and Meta-Analyses) [53]. The PRISMA checklist is provided in the Supplementary Materials (Document S1). The systematic review was prospectively registered in PROSPERO (ID: CRD420251129003). This Methods section outlines the systematic review process, eligibility criteria, search strategies, study selection, data extraction, quality assessment, and statistical analyses used, per PRISMA.

### 2.1. Eligibility Criteria

In accordance with the PRISMA guidelines [53], specific eligibility criteria were established. Eligible studies included intervention studies of any design involving individuals diagnosed with Autism Spectrum Disorder that investigated the effects of combined administration of omega-3 and vitamin D supplementation. Studies were required to report outcomes related to the improvement of emotional dysregulation symptoms associated with ASD. Only papers published in peer-reviewed academic journals were included; all publications had to be written in English and available in full text. To ensure the inclusion of up-to-date and relevant evidence, the search was limited to studies published from 2015 onwards. The 2015–2025 timeframe was selected to align the evidence base with the period following the release of DSM-5, during which diagnostic criteria for autism spectrum disorders and protocols for vitamin D and omega-3/6 supplementation have progressively converged toward greater standardization. This temporal boundary was intended to limit methodological heterogeneity and enhance comparability across studies.

### 2.2. Search Strategy

Two search strategies were employed: (1) a systematic search with predefined search string in major bibliographic databases, and (2) a snowball method to identify additional studies through the reference lists of all selected articles.

The search was performed in May 2025 across multiple scientific databases, including Cochrane Library, EBSCO_CINAHL, EBSCO_HRPC, EBSCO_PACD, Embase, Medline, PubMed, Scopus, Taylor & Francis, and Web of Science. The search string was designed through an iterative process, guided by a comprehensive overview of the available scientific literature. The string was formulated to include four main thematic domains: vitamin D, omega-3 fatty acids, nutritional supplementation, and autism spectrum disorder, specifically in terms of symptomatology for emotional and behavioral dysregulation.

The complete search string was:

((“Vitamin D” OR “cholecalciferol” OR “ergocalciferol”) AND (“omega-3” OR “omega 3 fatty acids” OR “n-3 fatty acids” OR “EPA” OR “DHA” OR “fish oil”) AND (“supplementation” OR “dietary supplements” OR “nutritional supplementation” OR “nutrient supplementation”) AND (“autism spectrum disorder” OR “Autism” OR “ASD” OR “autistic disorder” OR “pervasive developmental disorder”) AND (“behavioral dysregulation” OR “behavioral functioning” OR “emotional dysregulation” OR “aggression” OR “irritability” OR “tantrums” OR “hyperactivity” OR “externalizing behavior” OR “emotional symptoms”)).

### 2.3. Selection of Studies

**Figure 1:**
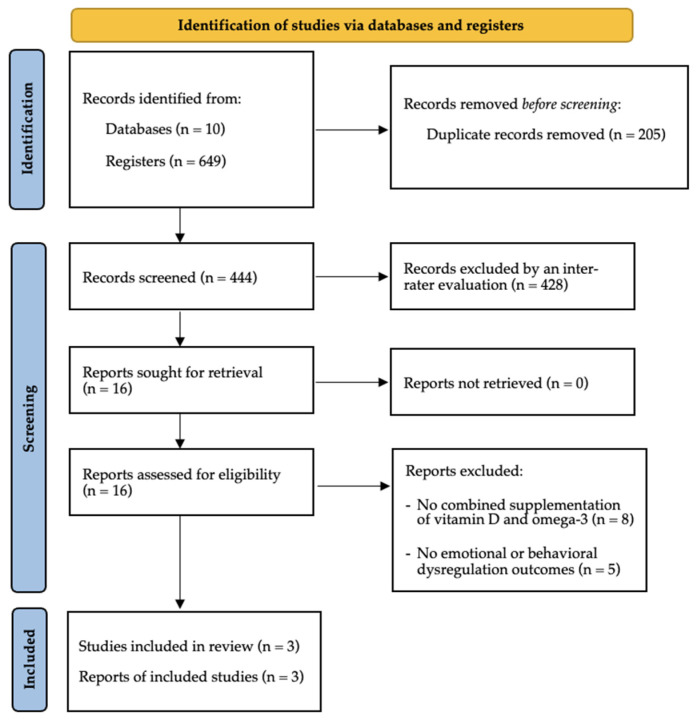
PRISMA Flow Diagram Showing Study Selection.

The results of searches are shown in the PRISMA diagram [53]. Originally, a total of 649 abstracts were identified, of which 205 duplicates were subsequently removed. The remaining 444 records were screened independently by two evaluators (M.B. and G.M.) based on titles and abstracts. Discrepancies were resolved by discussion, and both the reasons for inclusion and exclusion of each study were considered, leading to 100% inter-rater agreement. The 428 excluded records were removed for the following reasons: absence of combined vitamin D and omega-3 supplementation, lack of ASD samples, absence of emotional or behavioral outcomes, non-original articles including reviews, protocols, or conference abstracts, and studies not available in English. For the formal selection of studies, a third evaluator (S.C.) participated where needed to resolve disagreements. Inter-rater reliability, assessed using Cohen’s kappa coefficient, was 0.85, demonstrating high level agreement. After this procedure, three studies were identified that satisfied the specific inclusion criteria. Additional studies were sought through snowballing, but no further eligible papers were identified. As a result of the overall selection process, three studies were included, as shown in Figure 1.

### 2.4. Data Extraction

To perform systematic data extraction, an Excel spreadsheet was utilized for the selected studies. Data extraction was performed independently by two assessors who resolved discrepancies by consensus. The title of the study, year of publication, authors, journal, study design, objectives, sample size, participant gender and mean age, inclusion and exclusion criteria, assessment measures, statistical analyses, supplement dose, side effects, significant results, and major limitations of study data were recorded.

### 2.5. Quality Assessment

The methodological quality of the included studies was rated using design-specific appraisal tools. The current version of Risk of Bias 2 (RoB 2) tool was used for the randomized controlled trial [49], the Risk Of Bias In Non-randomized Studies—of Interventions, Version 2 (ROBINS-I V2) tool was used for the non-randomized study [54], while the Joanna Briggs Institute (JBI) Critical Appraisal Checklist for Case Reports was utilized for the case report [39]. The Results section presents the quality appraisal findings for each study.

## 3. Results

The characteristics of included studies are summarized in Table 1.

Three studies met the inclusion criteria and were included in the final analysis. These studies were published between 2019 and 2022 in peer-reviewed international journals (Nutritional Neuroscience, Journal of Steroid Biochemistry and Molecular Biology, and Metabolites). Although differing in design, all studies investigated nutritional supplementation with a particular emphasis on vitamin D and omega-3 fatty acids and their effects on emotional and behavioral outcomes in individuals with Autism Spectrum Disorders.

### 3.1. Study Characteristics

The studies were carried out in Italy and the United States, New Zealand, and Poland. Designs included one Randomized Controlled Trial (RCT) [52], one case report [54], and one observational cross-sectional analysis [53]. The age range of the sample sizes, which varied from one adult to 129 children, was 2.5–23 years. Every participant had an ASD diagnosis that was confirmed by the DSM-IV or DSM-5.

### 3.2. Intervention Characteristics

All studies investigated nutritional interventions involving vitamin D, omega-3 fatty acids, or broader multinutrient supplementation.

Participants in the RCT (*n* = 117; [49]) received vitamin D3 (2000 IU), omega-3 DHA (722 mg), or both daily for 12 months. A 23-year-old man in the case report (*n* = 1; [39]), received vitamin D3 (25,000 IU weekly) combined with omega-3 PUFAs (2.4 g EPA + 1.2 g DHA daily) for a duration of 24 months. The observational study (*n* = 129; [54]) compared children with ASD who were regularly supplemented with vitamin B complex, vitamin D3, omega-3 and omega-6 fatty acids, and probiotics, to their unsupplemented peers.

Supplementation was well tolerated in every study, and no negative side effects were noted.

### 3.3. Outcome Measures

Behavioral and emotional regulation outcomes were assessed using validated instruments, including the Childhood Autism Rating Scale (CARS), the Clinical Global Impressions (CGI) scale, and the Aberrant Behavior Checklist (ABC), particularly focusing on irritability and hyperactivity domains.

Nutritional and biochemical assessments included serum 25-hydroxyvitamin D [25(OH)D] concentrations, omega-3 indices (EPA + DHA percentage in red blood cells), and ratios of arachidonic acid to eicosapentaenoic acid (AA/EPA), as well as urinary concentrations of catecholamine metabolites—homovanillic acid (HVA) and vanillylmandelic acid (VMA), which serve as indicators of dopaminergic and noradrenergic activity.

### 3.4. Main Findings

Nutritional supplementation was found to have positive effects on behavioral and biochemical outcomes related to ASD in all three studies. 

In the case report [39], after two years of treatment, combined omega-3 and vitamin D supplementation led to a progressive improvement in irritability, agitation, and self-injurious behavior, with CARS scores decreasing from 38 to 33.5 and the AA/EPA ratio markedly improving from 49.54 to 1.96.

The RCT [49] demonstrated statistically significant reductions in irritability and hyperactivity among children receiving vitamin D and/or omega-3 supplementation compared to placebo (*p* < 0.05), accompanied by significant increases in serum 25(OH)D and omega-3 indices.

Finally, the cross-sectional study [54] reported lower urinary levels of HVA and VMA and behavioral improvements in children receiving a multicomponent supplement regimen. However, these effects cannot be attributed specifically to vitamin D and omega-3 and omega-6 supplementation, as the intervention included other additional nutrients (vitamin B complex, vitamin C and probiotics).

### 3.5. Quality Assessment Findings

The RCT was concluded to be of reasonably low risk of bias of randomization, blinding, and outcome as determined that randomization, blinding, and measure-benefit were all risk-free. An absence of conscious intention-to-treat analysis was an apparent source of possible bias. On balance, the study’s use of the trial’s methodology was acceptable, and its results were considered reasonably reliable. The observational study in [54], assessed based on ROBINS-I, demonstrated medium to high potential risk of bias because of its non-randomized design, absence of a randomized control group and interdependent supplementation dose variability. Although objective criteria for the biochemical outcome were based on standardized assessments, the behavioral measures were subjective, and the presence of uncontrolled confounders was not ruled out. These considerations seem to indicate that the results should be interpreted with caution. The case report [39], assessed via the JBI checklist, fulfilled critical methodological quality criteria, including an in-depth patient description, thorough documentation of the intervention, and well-reported outcomes. The follow-up was adequate, and the treatment implications section continued consistent with the literature. Nevertheless, the limitation of a single-case design, the lack of controls, susceptibility to observer and expectancy bias, and concurrent treatments detract from the generalizability of the findings.

## 4. Discussion

This systematic review explored the impact of combined vitamin D and omega-3 supplementation on symptoms of emotional and behavioral dysregulation in individuals with Autism Spectrum Disorder.

It is becoming more widely acknowledged that emotional dysregulation, which is characterized by irritability, affective lability, and difficulties controlling behavior under stress, is a key component of psychopathology in ASD and a significant cause of functional impairment [4,5]. This aligns with the RDoC framework, which was developed by the National Institute of Mental Health (NIMH) in 2009 [55], which conceptualizes emotional dysregulation as a transdiagnostic domain that involves alterations in the neural systems responsible for affective arousal, regulation, and stress response [56].

Across the studies included in this review [39,49,54], combined vitamin D and omega-3 supplementation was associated with improvements in emotional and behavioral regulation, although the evidence remains preliminary. In the randomized controlled trial of Mazahery et al. [49], daily intake of vitamin D3 (2000 IU) and omega-3 DHA (722 mg) resulted in significant reductions in irritability, whereas the increase in serum levels of vitamin D was accompanied by reductions in hyperactivity in children with ASD. Notably, this behavioral change paralleled increases in serum 25(OH)D as well as increases in omega-3 indices, indicating biological interaction with the intervention. Given the inherent limitations of single-case designs, supporting findings from Infante et al. [39] showed long-term improvements in irritability and self-injurious behaviors in a young adult after long-term co-supplementation along with normalization of the AA/EPA ratio and subsequent systemic inflammation-related response. Likewise, Gątarek & Kałużna-Czaplińska [54] showed reduced urinary levels of both HVA and VMA, indicators of dopaminergic and noradrenergic metabolism and lower reported behavioral symptoms among children receiving vitamin D, omega-3, omega-6 fats, and probiotics [54].

### 4.1. Biological Mechanisms and Potential Synergistic Interactions Between Vitamin D and Omega-3

Taken together, these results suggest that vitamin D and omega-3 may modulate neurochemical and inflammatory pathways relevant to emotional regulation and behavioral control. Both vitamin D and omega-3 fatty acids play pivotal roles in brain development, neurotransmission, and immune modulation, all of which are critical processes of emotional regulation. Vitamin D functions as a neurosteroid that stimulates neuronal differentiation and synaptogenesis, as well as modulates the synthesis of serotonin, dopamine, and GABA [34,57]. Its receptors and activating enzymes are expressed in large amounts in areas that are essential for affective processing, including the prefrontal cortex, hippocampus, and amygdala, and deficiency in vitamin D has been linked to increased inflammatory signaling, oxidative stress, and altered dopamine metabolism, processes that can exacerbate emotional reactivity [58].

Omega-3 fatty acids, particularly EPA and DHA, possess complementary effects by promoting neuronal membrane fluidity, receptor function, and neuroplasticity, while generating specialized mediators against neuroinflammation [59]. These properties are particularly relevant in ASD, where elevated cytokine levels, oxidative imbalance, and excitatory–inhibitory dysregulation are frequently reported [40].

Furthermore, emerging evidence also indicates reciprocal interactions between vitamin D and omega-3 pathways, particularly in neuroinflammatory and neuromodulatory processes [60], although clinical evidence of synergistic effects on emotional outcomes remains limited. Both nutrients may additionally modulate Hypothalamic–Pituitary–Adrenal (HPA) axis activity, potentially reducing stress-related affective responses associated with irritability and mood dysregulation in ASD [6,15].

### 4.2. Clinical Implications

The behavioral changes observed in all studies can be interpreted not merely as reductions in disruptive behavior, but as modulation of the core processes underlying emotional dysregulation. By attenuating neuroinflammation, while enhancing monoaminergic signaling and stabilizing stress reactivity, supplementation with vitamin D and omega-3 may contribute to the restoration of emotional homeostasis. This mechanism framework aligns with a broader shift in autism research: moving from symptom suppression toward interventions that target the biological underpinnings of self-regulation and emotional control [61]. Traditional pharmacological approaches, such as atypical antipsychotics, can mitigate irritability but often lead to side metabolic (i.e., weight gain, insulin resistance, dyslipidemia, hyperglycemia, and hypertension) and neurological (i.e., parkinsonism, akathisia, tardive dyskinesia and dystonia) effects, highlighting the need to explore complementary or adjunctive therapeutic strategies [62,63,64]. In contrast, nutritional modulation offers a physiologically coherent and low-risk alternative, particularly suited for long-term management. The safety and accessibility of vitamin D and omega-3 supplementation make these nutrients attractive as adjunctive treatments for emotional and behavioral dysregulation, potentially reducing reliance on pharmacotherapy. However, despite these encouraging results, the evidence base remains limited and heterogeneous. Differences in dosage (vitamin D from 2000 IU/day to 25,000 IU weekly; omega-3 from 722 mg DHA/day to 3.6 g EPA + DHA/day), duration, and outcome measures complicate comparisons. Emotion-related outcomes were often secondary endpoints, and only one study employed a fully randomized, placebo-controlled study design. Confounding factors such as baseline nutrient status and diet were not always controlled. Additionally, the generalizability of findings is limited by small, demographically narrow samples and the absence of long-term follow-up data.

The broader context of CAM in autism underscores the importance of strengthening the evidence base for nutritional and other adjunctive interventions. In a recent umbrella review, Gosling et al. [65] noted that while CAM approaches are widely used among autistic individuals, most remain supported by limited or low-quality evidence. Polyunsaturated fatty acids (PUFAs) were among the few interventions showing moderate support for safety and limited behavioral efficacy, consistent with the current findings. The methodological issues identified in that review, namely small samples, limited blinding, and inconsistent outcome reporting, mirror those seen in vitamin D and omega-3 research. To address these limitations, future studies should emphasize methodological rigor through large-scale, multi-center randomized controlled trials with clearly defined biochemical and behavioral endpoints. The use of validated instruments specifically designed to assess emotional dysregulation, such as the Emotion Dysregulation Inventory [66], would allow for more direct and standardized evaluation of treatment effects. Stratifying participants according to baseline inflammatory or nutritional markers could further clarify which subgroups are most responsive to intervention. Embedding such trials within structured evidence frameworks for complementary medicine could enhance transparency, reproducibility, and comparability across studies. Future studies should also disentangle the specific contribution of co-administered agents, such as other vitamins, probiotics or omega-6 fatty acids, to isolate the effects of vitamin D and omega-3.

## 5. Limitations

A key limitation of the present review is the small number of eligible studies, reflecting the limited body of research specifically addressing the combined supplementation of vitamin D and omega-3 fatty acids in relation to emotional dysregulation in individuals with ASD. Although only three studies met the inclusion criteria, it should be noted that there is no established minimum number of studies required for a systematic review. From a methodological standpoint, the majority of published reviews typically include at least two studies [67]. Moreover, the included studies exhibit considerable heterogeneity with respect to study design, intervention characteristics, and outcome assessment, which restricts the direct comparison of results. Taken together, the limited number of studies, along with variability in samples and methodologies, reduces the overall generalizability of the findings.

## 6. Conclusions

In conclusion, current limited evidence supports the hypothesis that combined vitamin D and omega-3 supplementation may beneficially modulate emotional and behavioral dysregulation in ASD by acting on shared pathways of inflammation, neurotransmission, and neuroendocrine regulation. Although current findings remain preliminary, the biological plausibility, safety, and accessibility of these interventions make them promising candidates for inclusion in multimodal strategies to improve emotional regulation in autism. Future trials should systematically assess baseline nutritional and inflammatory biomarkers to identify potential responders and non-responders, addressing a critical gap in the current literature. Establishing efficacy through rigorous, biomarker-informed randomized trials will be essential to confirm clinical value and to ultimately define the role of these interventions within personalized, nutritionally informed models of care.

## Figures and Tables

**Table 1:** The characteristics of the included studies.

	Intervention Type	Title (RIF.TO)	Study Design	Aim	Sample Size (M, F)	Age Range	Intervention Time	Measurement Assessment Emotional Dysregulation	Measurement Assessment Nutrients	Experimental Supplements	Supplement Dosage	Results (Relevant for the Review)	Side Effect	Main Findings
1	Omega-3 polyunsaturated fatty acids (PUFAs) and vitamin D co-supplementation	Omega-3 PUFAs and vitamin D co-supplementation as a safe-effective therapeutic approach for core symptoms of autism spectrum disorder: case report and literature review (Infante et al., 2020) [39]	Case report	To evaluate the therapeutic effect of combined omega-3 PUFA and vitamin D supplementation in a patient with ASD and review relevant literature	1 male	23 years	24 months	Childhood Autism Rating Scale (CARS), Clinical Global Impressions (CGI)	Serum 25(OH)D, AA/EPA ratio via gas chromatography	Cholecalciferol (vitamin D3) + ultra-refined omega-3 EPA + DHA concentrate	Vitamin D: 25,000 IU weekly; Omega-3: 2.4 g EPA + 1.2 g DHA/day	Marked improvement in irritability, agitation, and self-injurious behavior; CARS score decreased from 38 to 33.5; AA/EPA ratio decreased from 49.54 to 1.96	None reported	Omega-3 and vitamin D co-supplementation may safely and effectively reduce ASD core symptoms
2	Vitamin D and omega-3 long-chain polyunsaturated fatty acids (LCPUFA; DHA)	A randomized controlled trial of vitamin D and omega-3 long chain polyunsaturated fatty acids in the treatment of irritability and hyperactivity among children with autism spectrum disorder (Mazahery et al., 2019) [49]	Randomized, double-blind, placebo-controlled trial	To assess whether vitamin D, omega-3 LCPUFA, or their combination reduce irritability and hyperactivity in children with ASD	117 children (73 completers, sex not specified)	2.5–8 years	12 months	Aberrant Behavior Checklist (ABC)—Irritability and Hyperactivity subscales	Serum 25(OH)D and omega-3 index (EPA + DHA% RBC membranes)	Vitamin D3 (2000 IU/day), omega-3 LCPUFA (722 mg DHA/day), or both	Vitamin D: 2000 IU/day; Omega-3: 722 mg DHA/day	Significant reduction in irritability and hyperactivity; improved serum 25(OH)D and omega-3 indices; no severe adverse events	None serious; good compliance and tolerance	Vitamin D and omega-3 improved irritability; vitamin D also reduced hyperactivity
3	Vitamin B complex, Vitamin C, Vitamin D3, omega-3 and omega-6 fatty acids, probiotics	Effect of Supplementation on Levels of Homovanillic and Vanillylmandelic Acids in Children with Autism Spectrum Disorders (Gątarek & Kałużna-Czaplińska, 2022) [54]	Observational cross-sectional study with biochemical analysis	To determine how supplementation affects urinary levels of HVA and VMA and behavior in ASD children	129 children (sex not specified)	3–18 years	Cross-sectional (no fixed duration)	Parent questionnaires on behavioral changes	Urinary HVA and VMA measured by GC–MS	Vitamin B1, B3, B6, B12, C, D3, omega-3 and omega-6 fatty acids, probiotics	Not specified; based on caregiver reports	Supplemented children had lower urinary HVA and VMA compared with non-supplemented; behavioral improvement observed	None reported	Nutritional supplementation may modulate neurotransmitter metabolites linked to behavior in ASD

## Data Availability

No new data were created or analyzed in this study. Data sharing is not applicable to this article.

## Supplementary Materials

The following supporting information can be downloaded at: https://www.mdpi.com/article/10.3390/jcm15020745/s1, Document S1: The PRISMA checklist [68].
